# *TYK2* :p.Pro1104Ala Variant Protects Against Autoimmunity by Modulating Immune Cell Levels

**DOI:** 10.1111/imm.13902

**Published:** 2025-01-21

**Authors:** Maristella Steri, Valeria Orrù, Carlo Sidore, Antonella Mulas, Maristella Pitzalis, Fabio Busonero, Andrea Maschio, Valentina Serra, Mariano Dei, Sandra Lai, Francesca Virdis, Monia Lobina, Annalisa Loizedda, Michele Marongiu, Marco Masala, Matteo Floris, Nicolò Curreli, Lenuta Balaci, Francesco Loi, Maria Grazia Pilia, Alessandro Delitala, Edoardo Fiorillo, David Schlessinger, Magdalena Zoledziewska

**Affiliations:** ^1^ Institute of Genetic and Biomedical Research (IRGB) Italian National Research Council (CNR) Monserrato Sardinia Italy; ^2^ Department of Biomedical Sciences University of Sassari Sassari Italy; ^3^ Department of Medicine, Surgery and Pharmacy University of Sassari Sassari Italy; ^4^ Laboratory of Genetics and Genomics, National Institute on Aging (NIA) National Institutes of Health (NIH) Baltimore Maryland USA

**Keywords:** autoimmunity, immune cell levels, immunophenotyping, naïve cells, TYK2

## Abstract

The *TYK2*:p.Pro1104Ala (rs34536443) hypomorph variant has been associated with protection against numerous autoimmune disorders. Thus, its mechanism of action becomes of great interest. Here, consistent with the participation of activated immune cells in autoimmunity, we show that the variant regulates the levels of immune cells at a human, general population level and is associated particularly with higher levels of T and B lymphocytes, especially the naïve (non‐activated) compartment. Also, consistent with a protective function in autoimmunity, the level of regulatory CD4+ T cells was increased. Thus, this variant decreases immune activation thereby protecting from autoimmunity. Our work links the cellular mechanism regulated by the *TYK2*:p.Pro1104Ala variant to autoimmunity protection and supports TYK2 as a therapeutic target in autoimmunity.

## Graphical abstract

*TYK2*:p.Pro1104Ala variant confers protection from several autoimmune disorders but predisposes to infection and we show that the mechanism of its action includes an increase of the levels of lymphocytes in all compartments (CD4+, CD8+ and B), but these cells are mainly naive. Thus, this variant constrains immune activation thereby protecting from autoimmunity. Our work identifies the cellular mechanisms of regulation by the *TYK2*:p.Pro1104Ala variant providing novel hypotheses for the general immune mechanisms of protection from autoimmunity.

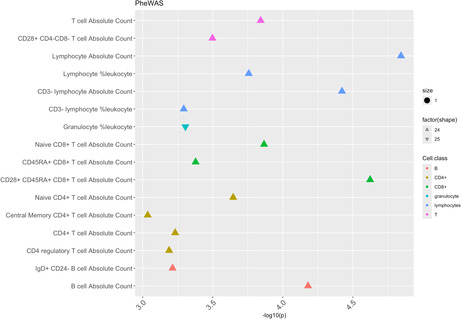

**Abbreviations**
FACS: fluorescence‐activated cell sorting
GWAS: Genome Wide Association Studies
IFN: interferon
JAK2: Janus kinase 2
MAF: minor allele frequency
MS: multiple sclerosis
RA: rheumatoid arthritis
STAT4: signal transducer and activator transcription 4
T1D: type 1 diabetes
TYK2: tyrosine kinase 2

## 1 Introduction

The immune response is characterised by extreme diversity. Part of that variability was established during the evolutionary history of populations and is genetically encoded.

GWAS studies have in fact revealed many variants affecting levels of immunity and/or autoimmunity. However, the mechanistic importance of such variability remains unknown. One such variant, *TYK2*:p.Pro1104Ala is associated with a lowered risk of several autoimmune disorders, and we have analysed its effects on immune cell levels [1, 2, 3, 4, 5, 6, 7, 8, 9].

Tyrosine kinase 2, encoded by the *TYK2* gene, is expressed ubiquitously and participates in the signalling of various antiviral and immunoregulatory cytokines (type I and type III IFNs, IL10, IL12, IL22 and IL23) with an impact both on immune and non‐immune cells [10, 11]. The targeted cytokine pathways are directly involved in immune‐mediated disorders.

Expressed in immune cells, TYK2 pairs with JAK2 and engages mainly with STAT4 to mediate signal transduction from IL12. This leads to T‐bet expression that promotes differentiation of naïve CD4+T cells into Th1 cells [12]. The TYK2/JAK2 heterodimer, acting through STAT1‐3, is further required for IL23 signalling, which regulates the differentiation and survival of Th17 cells [13]. In addition, TYK2 also combines with JAK1 and engages STAT1‐2 to mediate signalling of the potent antiviral type 1 IFNs and enhance various proinflammatory immune processes involving dendritic cells, macrophages, regulatory T cell differentiation and function, B cell activation and antibody production [14].

Several *TYK2* variants have been associated with autoimmunity and are generally protective [15]. In particular, loss of function mutations and inhibition of *TYK2* suppress autoimmunity in mice and humans [9, 16, 17, 18]. The *TYK2*:p.Pro1104Ala variant notably confers protection against autoinflammation in more than 10 autoimmune diseases including autoimmune thyroid disease, ankylosing spondylitis, Crohn's disease, psoriasis, systemic lupus erythematosus, rheumatoid arthritis (RA), sarcoidosis, systemic lupus erythematosus, type 1 diabetes (T1D), multiple sclerosis (MS), inflammatory bowel disease and ulcerative colitis [1, 2, 3, 4, 5, 6, 7, 8, 16]. The variant reduces the enzymatic activity of the corresponding protein [9, 16, 17]. Studies have suggested that low enzymatic activity is affected by stabilisation of an inactive conformation [18].

In agreement with the protection from autoimmunity provided by the *TYK2*:p.Pro1104Ala loss‐of‐function variant, selective *TYK2* inhibition has been intensively explored for the treatment of autoimmune diseases and several inhibitors have been developed [16, 19]. Although the mechanism of cellular regulation for this variant has been studied in patients with mycobacterial disease [20], the effect of *TYK2* variants on immune cell subpopulations has not been reported at a general, human population level. Here, we explore the regulatory effects of *TYK2*:p.Pro1104Ala variant on the levels of immune cells that underlie its regulation of the immune response.

## 2 Methodology

### 2.1 Population Cohort

We cross‐compared the *TYK2*:p.Pro1104Ala variant association with different autoimmune disorders with immunophenotyping associations in the SardiNIA cohort [21].

The SardiNIA general population cohort from the Lanusei valley area in Sardinia, Italy, has been previously described [22]. The cohort was phenotyped for over 1500 quantitative traits, including immune‐related phenotypes. Details of phenotype and genotype assessments for these samples have been published previously [23].

### 2.2 GWAS on Immune Phenotypes

For this study, we used GWAS data on 731 immune phenotypes measured in 3757 Sardinian individuals. The traits were measured by extended flow cytometry (FACS) profiling [23, 24]. FACS profiling on fresh blood samples was performed within 2 h of blood collection, as described previously [25]. Summary statistics for 731 immune traits in the region of the *TYK2* gene were assessed in the SardiNIA study. These traits include 118 absolute cell counts, 389 MFIs of surface antigens and 32 morphological parameters. The significance threshold has been calculated by correcting the nominal *p* of 0.05 for these 539 statistically independent traits, applying a multiple‐independent test (Bonferroni correction) and reaching a significant threshold of 9.28 × 10^−5^. The remaining 192 relative counts, corresponding to percentages with respect to hierarchically higher cell population, are instead statistically dependent on the absolute cell counts tested and were not considered for Bonferroni correction.

### 2.3 Colocalization Analysis

To determine whether a disease and an immune trait share the same genetic causal variant in the *TYK2* gene region, we performed a colocalization test in a region of about 50 kb around the *TYK2*:p.Pro1104Ala variant using the summary statistics for the counts of lymphocyte subpopulations in the SardiNIA cohort and publicly available summary statistics for T1D, RA and MS [26, 27, 28]. Two association signals were considered colocalizing if their posterior probability of a shared variant (PP.H4) was ≥ 0.8 [21]. Colocalization analysis was performed using the R ‘coloc’ package [29].

## 3 Results

### 3.1 *TYK2* :p.Pro1104Ala Variant Worldwide Frequencies

**FIGURE 1:**
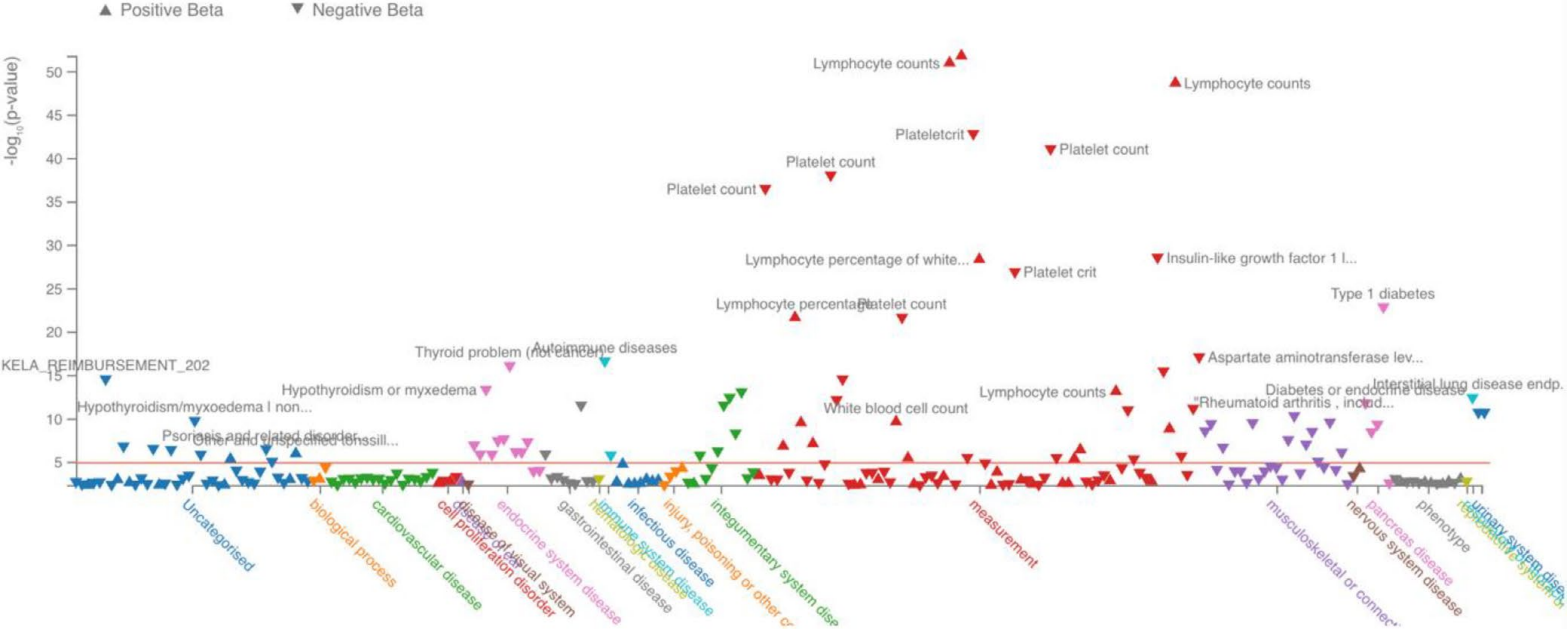
PheWAS data of traits associated with rs34536443 (TYK2) variant in the UK Biobank and GWAS Catalogue summary statistics (*p* < 0.05) according to the Open Targets Genetics database (https://genetics.opentargets.org/).

The *TYK2*:p.Pro1104Ala variant is frequent in European populations (MAF = 4.26%), less common in Sardinia (MAF = 2.79%), rare in African populations (MAF = 0.96%) and absent in East Asia. These observations are in agreement with the selective sweep described for this variant [30]. The *TYK2*:p.Pro1104Ala variant has a Combined Annotation Dependent Depletion score of 25.5, and is associated with several traits and conditions Figure 1, confirming its functional importance.

### 3.2 Effect of *TYK2* :p.Pro1104Ala on T Lymphocytes

**FIGURE 2:**
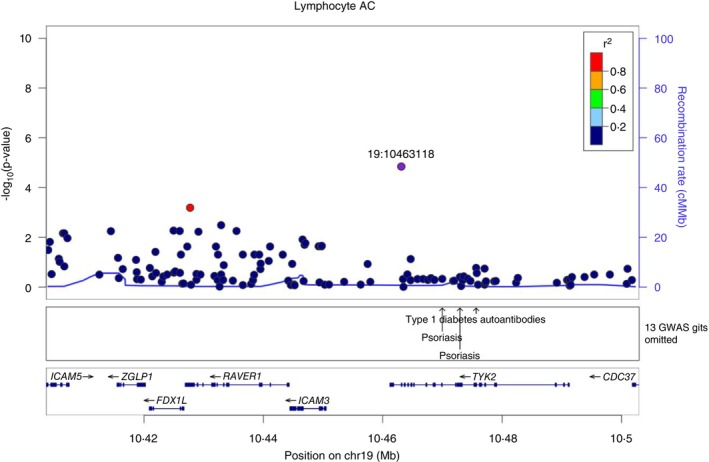
Regional plot of genetic association of the *TYK2* gene region with lymphocyte absolute count phenotype. The association strength (−log10(*p*); *y* axis) is plotted versus genomic positions (on the hg19/GRCh37 genomic build; *x* axis). SNPs are coloured to reflect their LD with rs34536443 (TYK2:p.Pro1104Ala) (which is indicated with a purple dot).

We show that rs34536443 (*TYK2*:p.Pro1104Ala), allele C which confers protection from different autoimmune disorders has been associated with several immunophenotypes. The most significantly associated cell population in our study is lymphocytes, which substantially increase (*p* = 1.43 × 10^−5^, beta = 0.325), Figure 2, Table 1. This result is in agreement with published observations for this variant [31, 32]. In addition, as representative examples of associated traits, we found a very high colocalization probability between the association of total lymphocyte counts in the SardiNIA cohort and T1D (PP.H4 = 96.6%), RA (97.5%) and MS (98.4%) (Figures S1 and S2).

**TABLE 1:** *TYK2*:p.Pro1104Ala (rs34536443) associations across immunophenotyped traits.

Trait	*N*	*p*	Effect (beta)	STERR
Lymphocyte AC	3653	1.43E‐05	0.325	0.074
Lymphocyte %leukocyte	3669	1.75E‐04	0.282	0.075
T cell AC	3653	1.44E‐04	0.280	0.074
CD4+ AC	3652	5.87E‐04	0.257	0.075
Naive CD4+ AC	3395	2.26E‐04	0.270	0.073
CD4 Treg AC	3405	6.48E‐04	0.265	0.078
CM CD4+ AC	3395	9.21E‐04	0.259	0.078
Naive CD8br AC	3395	1.36E‐04	0.200	0.052
CD45RA+ CD8br AC	3395	4.19E‐04	0.236	0.067
CD28+ CD45RA+ CD8br AC	3408	2.38E‐05	0.229	0.054
CD28+ DN (CD4−CD8−) AC	3408	3.18E‐04	0.240	0.067
CD3− lymphocyte %leukocyte	3669	5.10E‐04	0.264	0.076
CD3‐ lymphocyte AC	3653	3.78E‐05	0.309	0.075
B cell AC	3653	6.60E‐05	0.293	0.073
IgD+ CD24− AC	3656	6.12E‐04	0.264	0.077
Granulocyte %leukocyte	3669	4.96E‐04	−0.264	0.076

*Note*: Columns from left to right give the trait name, *p* of association, the effect size expressed in standard deviation units (effect), its standard error (STERR). The significant threshold of association *p* threshold is (*p* < 9.28E‐05).

Abbreviations: AC—absolute count; CM—central memory; DN—double negative; MAF—minor allele frequency.

In further analyses of lymphocyte compartment, T cell absolute count increases (*p* = 1.44 × 10^−4^, beta = 0.280), Table 1, and within the T cell compartment, CD4+ absolute count increases (*p* = 5.87 × 10^−4^, beta = 0.257), within which CD4+ naïve T cells increase (*p* = 2.26 × 10^−4^, beta = 0.27), Table 1. CD4+ regulatory T cells also suggestively increase (*p* = 6.48 × 10^−4^, beta = 0.265), which is in line with the protective function of regulatory T cells in autoimmunity, Table 1.

We also noted that the central memory CD4 reservoir for effector memory T cells increases (*p* = 9.21 × 10^−4^, beta = 0.259). Central memory CD4 T cells show enhanced proliferation. These cells synthesise IL2 and have less potential for rapid IFN‐gamma or IL4 secretion [33].

In the CD8 T cell compartment, the naïve CD28+ CD45RA+CD8br cell level significantly increased (*p* = 2.38 × 10^−5^, beta = 0.229). These cells have not yet encountered antigen and are therefore poorly differentiated. After activation, for example during chronic virus exposure, naïve CD8+ bystander cells differentiate into a memory‐like phenotype. Exposure of naïve CD8 T cells to type I interferons drive the rapid acquisition of effector function after antigenic stimulation [34].

Interestingly, we found a suggestive association with the CD28+ double negative (DN, CD4−CD8−) T cell population (*p* = 3.18 × 10^−4^, beta = 0.240), Table 1. DN T cells are a rare subset of peripheral T cells whose important role in inflammation, immune‐related diseases and cancer has been recently discussed [35]. Our data suggest a positive effect of CD28+DN T cells on autoimmunity, in line with a previous study in which these cells were described to have a regulatory function in non‐obese diabetic mouse (NOD) model [36]. DN regulatory T cells have the capacity to prevent graft‐versus‐host disease and have therapeutic value for autoimmune diseases depending on their regulatory effects on CD8+, CD4+ and B cells [35].

Additionally, CD3‐ lymphocytes are significantly increased (*p* = 3.78 × 10^−5^, beta = 0.309) suggesting an expansion of B cell and/or NK cell levels; indeed, we directly observed an increase in B cells (next paragraph and Table 1).

### 3.3 Effect of *TYK2* :p.Pro1104Ala on B Cells

One of the largest effects of the *TYK2*:p.Pro1104Ala mutation on immune cell levels is an increase in the absolute count of B cells (*p* = 6.60 × 10^−5^, beta = 0.293), Table 1. Within that population, the most associated are IgD+CD24− (*p* = 6.12 × 10^−4^, beta = 0.264) that mainly include mature naïve B cells [37].

### 3.4 Effect of *TYK2* :p.Pro1104Ala on Granulocytes

The granulocyte frequency with respect to leukocytes is the only subpopulation suggestively downregulated by the *TYK2*:p.Pro1104Ala variant (*p* = 4.96 × 10^−4^, beta = −0.264), Table 1. This may be a secondary effect, resulting from an alteration of the relative frequencies of cell subsets attendant on the increase of lymphocytes.

## 4 Discussion

The fundamental role of TYK2 in autoimmunity and immunodeficiencies is well‐established. Autosomal recessive, complete *TYK2* deficiency leads to immunodeficiency, while variants that reduce TYK2 signalling, including *TYK2*:p.Pro1104Ala, are associated with protection from autoimmunity. Consistent with those findings, *Tyk2−/−* mice do not develop experimental autoimmune encephalomyelitis, exhibit impaired polarisation of Th1 and Th2 and a reduction of IL‐17+IFN‐gamma T cells [38, 39]. Furthermore, the role of *TYK2* inhibition on pro‐inflammatory Th1 and Th2 has been described [20]. In this regard, the *TYK2*:p.Pro1104Ala loss‐of‐function variant has been associated with protection against autoimmunity [16]. Furthermore, we found that the *TYK2*:p.Pro1104Ala is associated with increased levels of T and B lymphocytes, especially the naïve compartment. These results are in agreement with the previously described findings that in autoimmunity naïve compartment contracts while effector memory cells expand [40, 41].

Although, the increased levels of lymphocytes have been observed in other studies [31, 32], and the effect of the *TYK2*:p.Pro1104Ala variant on the expansion of naïve T cells has been demonstrated in homozygous individuals with mycobacterial disease [20], to our knowledge no one has dissected, in detail the association of leukocyte subsets with this variant at a general, human population level. Additionally, our study reports the effects of the variant on the other previously unreported shifts in immune cell subpopulations, for example regulatory T cells and the double‐negative population, CD28+DN (CD4− CD8−). However, these novel findings require confirmation in other cohorts.

Our results explain why paradoxically, the *TYK2*:p.Pro1104Ala variant, in the context of its conferred protection against autoimmunity, is at the same time associated with an increased level of lymphocytes.

We observed an increase of central memory helper T cells that have little or no effector function, with a low‐activation threshold and high proliferation and differentiation in response to antigenic stimulation. Thus, they are cells arrested at intermediate stages of differentiation preceding effector memory T cells status. They may function as stem cells under homeostatic conditions [42]. This finding suggests that this variant exerts a different effect on individuals in the general population compared to the previously described decrease in this cell population in individuals with mycobacterial disease [20]. Thus, the immune milieu created by the *TYK2*:p.Pro1104Ala variant is not completely unrelated to a proper immune response and we suggest that the shift in memory helper T cells may have a role in protection from autoimmunity, but this observation requires replication in independent studies.

Furthermore, we observed that *TYK2*:p.Pro1104Ala variant mediates an increase in CD3‐lymphocytes. Previous studies have demonstrated that, in wild‐type mice, CD3‐negative cells (comprising NK and B cells) produce small amounts of IFN‐gamma, in contrast CD3‐negative cells from Tyk2‐deficient mice did not produce IFN‐gamma [43], a mechanism that may ulteriorly contribute to the protection.

Additionally, for first time we show that *TYK2*:p.Pro1104Ala variant increases the regulatory CD4+ T cells, in line with the protective function of this variant in autoimmunity [44]. Consistently with this finding BMS‐986202 selective *TYK2* inhibitor redirected CD4+T cells toward a regulatory T cell phenotype [45].

Moreover, considering the cytotoxic T cell compartment, the level of CD28+CD45RA+CD8br naïve cells significantly increase. These are naïve CD8+ bystander cells that have not encountered antigens and, after activation by pathogens like viruses, can differentiate into a memory‐like phenotype [34]. Exposure of naïve CD8 T cells to type I interferons causes fast transformation of these cells to effector cells, and still provides a proper response to viral infection even in carriers of the *TYK2*:p.Pro1104Ala variant [16, 38, 46].

A recent study demonstrated that TYK2 signalling promotes the development of autoreactive CD8+ cytotoxic lymphocytes in autoimmune diabetes. The loss of Tyk2 inhibits the development of the autoreactive CD8+T‐BET+ cytotoxic lymphocytes and treatment with BMS‐986165 a selective TYK2 inhibitor, inhibits the expansion of T‐BET+CTLs [46].

We found another factor in the protection by *TYK2*:p.Pro1104Ala variant: the suggestive association of DN population CD28+DN (CD4− CD8−). DN T cells are considered disease‐causing and reduced or absent expression of CD28 appears to be characteristic for most of the peripheral blood DN cells; they are considered antigen‐experienced and differentiated [47]. CD28 is involved in proper differentiation of DN and B7‐CD28 interaction promotes proliferation and survival but suppresses differentiation of DN T cells in thymus [48]. It has been known that the majority of DN T cells that contribute to the autoimmune and autoinflammatory disorders exhibit effector phenotypes and thus, we hypothesized that the *TYK2*:p.Pro1104Ala mutation may suppress DN differentiation, instead promotes differentiation of DN thymocytes into double positive and then regulatory T cells, thus protects from the disease guided by CD28 [49]. Our data suggest a positive effect of CD28+ subpopulations in protecting from autoimmunity, indeed CD28 co‐stimulation provides a proper T cell activation promoting cell cycle entry and the production of various cytokines [50].

One of the other largest effects of *TYK2*:p.Pro1104Ala mutation on immune cell levels was an increase in the absolute count of B cells, most notably the IgD+CD24− mature naïve B cells population increased, confirming the protective effect of naïve cells on autoimmunity.

Our data are in agreement with a recent study that demonstrated that JAK–STAT signalling, apart from its role in immune responses, is a major regulator of immune cell homeostasis, preparing cells for a rapid response to immune stimuli [51].

Conceivably, autosomal recessive complete *TYK2* deficiency predisposes to severe recurrent infections [52]. Additionally, the *TYK2*:p.Pro1104Ala variant selectively impairs cellular responses to IL23, but not to IFN‐alpha or IL10, in agreement with its predisposing effect for tuberculosis [20, 53]. Furthermore, healthy individuals with the protective variant decrease IFN I signalling and have a decreased frequency of circulating Tfh (T follicular helper) cells and switched memory B cells [38].

Additionally, the Tyk2 knock‐in murine model showed less IL12 receptor signalling and diminished in vitro Th1 skewing. Finally, T cells had reduced IL23‐dependent signalling and diminished ability to skew toward Th17 in vitro [38]. However, we do not exclude other effects of the *TYK2*:p.Pro1104Ala variant on immune system regulation.

Targeting *TYK2* has already been exploited clinically. One of the autoimmune diseases, psoriasis is largely an IL23‐driven disease, and thus inhibitors of TYK2 are effective therapy. Indeed, BMS‐986165 (Deucravacitinib) is a selective potent oral TYK2 inhibitor currently used for psoriasis treatment [54], and two other TYK2 inhibitors (TAK‐279, VTX958) are under development for psoriasis treatment. Deucravacitinib's mechanism of action is distinct from JAK inhibitors; it binds to an allosteric site rather than an ATP‐binding site, and its selectivity for TYK2 could potentially limit side effects [55]. Oral treatment with the selective *TYK2* inhibitor, BMS‐986165 was also efficacious and ameliorated progression in murine models of spondylarthritis, lupus nephritis, inflammatory bowel disease, and autoimmune diabetes [46, 56].

Treatment with BMS‐986165 has been shown to reduce the expansion of cytotoxic T lymphocytes. In line with that action, Tyk2 deficiency inhibits the development of autoreactive CD8+ T cells and increases inflammatory responses in beta‐cells during aging [46].

*TYK2* variants are associated with critical COVID‐19, and the *TYK2*:p.Pro1104Ala variant has been shown to be causal [57]. Interestingly, protection from severe COVID‐19 has been described in Sardinia [58]. Hypothetically, given the increased risk of severe COVID‐19 conferred by the *TYK2* variant, its lower frequency on the island could in part, together with other COVID‐19 protecting variants, contribute to the protection against severe disease but decrease the general protection from autoimmunity, known to be common in Sardinia.

However, there is no satisfactory explanation for how such a variant, which favours a less aggressive posture of the immune system, arose and persisted in the populations. In a general, variants that protect against the infection are under selective pressure and widespread in a population but at the cost of risk of autoimmunity.

In fact, the *TYK2*:p.Pro1104Ala variant is the main common risk factor for tuberculosis in endemic regions and increases the risk of severe COVID‐19 [16, 20, 59]. Carriers of the *TYK2*:p.Pro1104Ala variant are predisposed to tuberculosis, acting possibly through reduced IL23 and IL12 signalling‐dependent IFN‐gamma production [16, 20]. Heterozygous carriers have decreased numbers of follicular helper T cells and lower IFN‐signalling in naïve but not in effector cells [38]. Indeed, it has been estimated that this variant arose around 30 000 years ago and then underwent strong negative selection in Europeans over the past 2000 years, which has been inferred from the co‐occurrence of the emergence of *Mycobacterium tuberculosis* [60].

In attempting to understand the complex yin and yang of predisposition to infectious disease and protection from autoimmunity of the *TYK2*:p.Pro1104Ala variant, our study is a first step toward understanding of cellular mechanisms regulated by this variant.

## 5 Conclusion

For the first time, we describe that the *TYK2*:p.Pro1104Ala general autoimmunity protective variant exerts its function through effects on multiple immune cell subpopulations at a human, general population level. The effects of this variant are complex and includes changes additional to cytokine response. It increases the level of lymphocytes in all compartments (CD4+, CD8+ and B), but we confirmed that these cells are mainly the naïve populations that are particularly useful in fighting against autoimmunity. The protective character of the variant is further rationalised by an increase of T regulatory cells with anti‐autoimmune potential. Our work underlines the current utility of TYK2 as a therapeutic target in autoimmunity.

## Conflicts of Interest

C.S. is currently the employee of Regeneron Pharmaceuticals and beneficiary of stock options and grants in Regeneron. The other authors declare no conflicts of interest.

## Supporting information


Data S1.

## Data Availability

Data sharing not applicable to this article as no datasets were generated or analysed during the current study.

## References

[1] J. Chiou , R. J. Geusz , M.‐L. Okino , et al., “Interpreting Type 1 Diabetes Risk With Genetics and Single‐Cell Epigenomics,” Nature 594 (2021): 398–402, 10.1038/s41586-021-03552-w. 34012112 PMC10560508

[2] L. C. Tsoi , S. L. Spain , J. Knight , et al., “Identification of 15 New Psoriasis Susceptibility Loci Highlights the Role of Innate Immunity,” Nature Genetics 44 (2012): 1341–1348, 10.1038/ng.2467. 23143594 PMC3510312

[3] J. Bentham , D. L. Morris , D. S. C. Graham , et al., “Genetic Association Analyses Implicate Aberrant Regulation of Innate and Adaptive Immunity Genes in the Pathogenesis of Systemic Lupus Erythematosus,” Nature Genetics 47 (2015): 1457–1464, 10.1038/ng.3434. 26502338 PMC4668589

[4] V. Forgetta , D. Manousaki , R. Istomine , et al., “Rare Genetic Variants of Large Effect Influence Risk of Type 1 Diabetes,” Diabetes 69 (2020): 784–795, 10.2337/db19-0831. 32005708 PMC7085253

[5] D. Ellinghaus , L. Jostins , S. L. Spain , et al., “Analysis of Five Chronic Inflammatory Diseases Identifies 27 New Associations and Highlights Disease‐Specific Patterns at Shared Loci,” Nature Genetics 48 (2016): 510–518, 10.1038/ng.3528. 26974007 PMC4848113

[6] K. M. de Lange , L. Moutsianas , J. C. Lee , et al., “Genome‐Wide Association Study Implicates Immune Activation of Multiple Integrin Genes in Inflammatory Bowel Disease,” Nature Genetics 49 (2017): 256–261, 10.1038/ng.3760. 28067908 PMC5289481

[7] International Multiple Sclerosis Genetics Consortium , “Multiple Sclerosis Genomic Map Implicates Peripheral Immune Cells and Microglia in Susceptibility,” Science 365 (2019): eaav7188, 10.1126/science.aav7188. 31604244 PMC7241648

[8] A. Márquez , M. Kerick , A. Zhernakova , et al., “Meta‐Analysis of Immunochip Data of Four Autoimmune Diseases Reveals Novel Single‐Disease and Cross‐Phenotype Associations,” Genome Medicine 10 (2018): 97, 10.1186/s13073-018-0604-8. 30572963 PMC6302306

[9] N. Couturier , F. Bucciarelli , R. N. Nurtdinov , et al., “Tyrosine Kinase 2 Variant Influences T Lymphocyte Polarization and Multiple Sclerosis Susceptibility, Brain,” Journal of Neurology 134 (2011): 693–703, 10.1093/brain/awr010. 21354972

[10] B. Strobl , D. Stoiber , V. Sexl , and M. Mueller , “Tyrosine Kinase 2 (TYK2) in Cytokine Signalling and Host Immunity,” Frontiers in Bioscience (Landmark Edition) 16 (2011): 3214–3232, 10.2741/3908. 21622231

[11] N. R. Leitner , A. Witalisz‐Siepracka , B. Strobl , and M. Müller , “Tyrosine Kinase 2—Surveillant of Tumours and Bona Fide Oncogene,” Cytokine 89 (2017): 209–218, 10.1016/j.cyto.2015.10.015. 26631911

[12] E. D. Tait Wojno , C. A. Hunter , and J. S. Stumhofer , “The Immunobiology of the Interleukin‐12 Family: Room for Discovery,” Immunity 50 (2019): 851–870, 10.1016/j.immuni.2019.03.011. 30995503 PMC6472917

[13] T. Korn , E. Bettelli , M. Oukka , and V. K. Kuchroo , “IL‐17 and Th17 Cells,” Annual Review of Immunology 27 (2009): 485–517, 10.1146/annurev.immunol.021908.132710. 19132915

[14] G. R. Stark and J. E. Darnell , “The JAK‐STAT Pathway at Twenty,” Immunity 36 (2012): 503–514, 10.1016/j.immuni.2012.03.013. 22520844 PMC3909993

[15] Z. Li , M. Rotival , E. Patin , F. Michel , and S. Pellegrini , “Two Common Disease‐Associated TYK2 Variants Impact Exon Splicing and TYK2 Dosage,” PLoS One 15 (2020): e0225289, 10.1371/journal.pone.0225289. 31961910 PMC6974145

[16] C. A. Dendrou , A. Cortes , L. Shipman , et al., “Resolving TYK2 Locus Genotype‐To‐Phenotype Differences in Autoimmunity,” Science Translational Medicine 8 (2016): 363ra149, 10.1126/scitranslmed.aag1974. PMC5737835 27807284

[17] M. H. Tomasson , Z. Xiang , R. Walgren , et al., “Somatic Mutations and Germline Sequence Variants in the Expressed Tyrosine Kinase Genes of Patients With De Novo Acute Myeloid Leukemia,” Blood 111 (2008): 4797–4808, 10.1182/blood-2007-09-113027. 18270328 PMC2343607

[18] N. Lesgidou , E. Eliopoulos , G. N. Goulielmos , and M. Vlassi , “Insights on the Alteration of Functionality of a Tyrosine Kinase 2 Variant: A Molecular Dynamics Study,” Bioinformatics 34 (2018): i781–i786, 10.1093/bioinformatics/bty556. 30423093

[19] L. T. Jensen , K. E. Attfield , M. Feldmann , and L. Fugger , “Allosteric TYK2 Inhibition: Redefining Autoimmune Disease Therapy Beyond JAK1‐3 Inhibitors,” eBioMedicine 97 (2023): 104840, 10.1016/j.ebiom.2023.104840. 37863021 PMC10589750

[20] S. Boisson‐Dupuis , N. Ramirez‐Alejo , Z. Li , et al., “Tuberculosis and Impaired IL‐23‐Dependent IFN‐γ Immunity in Humans Homozygous for a Common TYK2 Missense Variant,” Science Immunology 3 (2018): eaau8714, 10.1126/sciimmunol.aau8714. 30578352 PMC6341984

[21] V. Orrù , M. Steri , C. Sidore , et al., “Complex Genetic Signatures in Immune Cells Underlie Autoimmunity and Inform Therapy,” Nature Genetics 52 (2020): 1036–1045, 10.1038/s41588-020-0684-4. 32929287 PMC8517961

[22] G. Pilia , W.‐M. Chen , A. Scuteri , et al., “Heritability of Cardiovascular and Personality Traits in 6,148 Sardinians,” PLoS Genetics 2 (2006): e132, 10.1371/journal.pgen.0020132. 16934002 PMC1557782

[23] C. Sidore , F. Busonero , A. Maschio , et al., “Genome Sequencing Elucidates Sardinian Genetic Architecture and Augments Association Analyses for Lipid and Blood Inflammatory Markers,” Nature Genetics 47 (2015): 1272–1281, 10.1038/ng.3368. 26366554 PMC4627508

[24] V. Orrù , M. Steri , G. Sole , et al., “Genetic Variants Regulating Immune Cell Levels in Health and Disease,” Cell 155 (2013): 242–256, 10.1016/j.cell.2013.08.041. 24074872 PMC5541764

[25] M. Steri , V. Orrù , M. L. Idda , et al., “Overexpression of the Cytokine BAFF and Autoimmunity Risk,” New England Journal of Medicine 376 (2017): 1615–1626, 10.1056/NEJMoa1610528. 28445677 PMC5605835

[26] J. C. Censin , C. Nowak , N. Cooper , P. Bergsten , J. A. Todd , and T. Fall , “Childhood Adiposity and Risk of Type 1 Diabetes: A Mendelian Randomization Study,” PLoS Medicine 14 (2017): e1002362, 10.1371/journal.pmed.1002362. 28763444 PMC5538636

[27] Y. Okada , D. Wu , G. Trynka , et al., “Genetics of Rheumatoid Arthritis Contributes to Biology and Drug Discovery,” Nature 506 (2014): 376–381, 10.1038/nature12873. 24390342 PMC3944098

[28] International Multiple Sclerosis Genetics Consortium (IMSGC) , A. H. Beecham , N. A. Patsopoulos , et al., “Analysis of Immune‐Related Loci Identifies 48 New Susceptibility Variants for Multiple Sclerosis,” Nature Genetics 45 (2013): 1353–1360, 10.1038/ng.2770. 24076602 PMC3832895

[29] C. Wallace , “A More Accurate Method for Colocalisation Analysis Allowing for Multiple Causal Variants,” PLoS Genetics 17 (2021): e1009440, 10.1371/journal.pgen.1009440. 34587156 PMC8504726

[30] G. Kerner , G. Laval , E. Patin , et al., “Human Ancient DNA Analyses Reveal the High Burden of Tuberculosis in Europeans Over the Last 2,000 Years,” American Journal of Human Genetics 108 (2021): 517–524, 10.1016/j.ajhg.2021.02.009. 33667394 PMC8008489

[31] D. Vuckovic , E. L. Bao , P. Akbari , et al., “The Polygenic and Monogenic Basis of Blood Traits and Diseases,” Cell 182 (2020): 1214–1231.e11, 10.1016/j.cell.2020.08.008. 32888494 PMC7482360

[32] M.‐H. Chen , L. M. Raffield , A. Mousas , et al., “Trans‐Ethnic and Ancestry‐Specific Blood‐Cell Genetics in 746,667 Individuals From 5 Global Populations,” Cell 182 (2020): 1198–1213.e14, 10.1016/j.cell.2020.06.045. 32888493 PMC7480402

[33] F. Sallusto , D. Lenig , R. Förster , M. Lipp , and A. Lanzavecchia , “Two Subsets of Memory T Lymphocytes With Distinct Homing Potentials and Effector Functions,” Nature 401 (1999): 708–712, 10.1038/44385. 10537110

[34] D. F. Tough , P. Borrow , and J. Sprent , “Induction of Bystander T Cell Proliferation by Viruses and Type I Interferon In Vivo,” Science 272 (1996): 1947–1950, 10.1126/science.272.5270.1947. 8658169

[35] Z. Wu , Y. Zheng , J. Sheng , et al., “CD3+CD4‐CD8‐ (Double‐Negative) T Cells in Inflammation, Immune Disorders and Cancer,” Frontiers in Immunology 13 (2022): 816005, 10.3389/fimmu.2022.816005. 35222392 PMC8866817

[36] B. Duncan , C. Nazarov–Stoica , J. Surls , et al., “Double Negative (CD3+4−8−) TCRαβ Splenic Cells From Young NOD Mice Provide Long‐Lasting Protection Against Type 1 Diabetes,” PLoS One 5 (2010): e11427, 10.1371/journal.pone.0011427. 20625402 PMC2896421

[37] F. F. K. Mensah , C. W. Armstrong , V. Reddy , et al., “CD24 Expression and B Cell Maturation Shows a Novel Link With Energy Metabolism: Potential Implications for Patients With Myalgic Encephalomyelitis/Chronic Fatigue Syndrome,” Frontiers in Immunology 9 (2018): 2421, 10.3389/fimmu.2018.02421. 30405620 PMC6204382

[38] J. A. Gorman , C. Hundhausen , M. Kinsman , et al., “The TYK2‐P1104A Autoimmune Protective Variant Limits Coordinate Signals Required to Generate Specialized T Cell Subsets,” Frontiers in Immunology 10 (2019): 44, 10.3389/fimmu.2019.00044. 30740104 PMC6355696

[39] A. Oyamada , H. Ikebe , M. Itsumi , et al., “Tyrosine Kinase 2 Plays Critical Roles in the Pathogenic CD4 T Cell Responses for the Development of Experimental Autoimmune Encephalomyelitis,” Journal of Immunology 183 (2009): 7539–7546, 10.4049/jimmunol.0902740. 19917699

[40] I. Raphael , R. R. Joern , and T. G. Forsthuber , “Memory CD4+ T Cells in Immunity and Autoimmune Diseases,” Cells 9 (2020): 531, 10.3390/cells9030531. 32106536 PMC7140455

[41] P. Bhargava and P. A. Calabresi , “Novel Therapies for Memory Cells in Autoimmune Diseases,” Clinical and Experimental Immunology 180 (2015): 353–360, 10.1111/cei.12602. 25682849 PMC4449764

[42] F. Sallusto , J. Geginat , and A. Lanzavecchia , “Central Memory and Effector Memory T Cell Subsets: Function, Generation, and Maintenance,” Annual Review of Immunology 22 (2004): 745–763, 10.1146/annurev.immunol.22.012703.104702. 15032595

[43] K. Shimoda , K. Kato , K. Aoki , et al., “Tyk2 Plays a Restricted Role in IFN Alpha Signaling, Although It Is Required for IL‐12‐Mediated T Cell Function,” Immunity 13 (2000): 561–571, 10.1016/s1074-7613(00)00055-8. 11070174

[44] S. Sakaguchi , N. Mikami , J. B. Wing , A. Tanaka , K. Ichiyama , and N. Ohkura , “Regulatory T Cells and Human Disease,” Annual Review of Immunology 38 (2020): 541–566, 10.1146/annurev-immunol-042718-041717. 32017635

[45] K. Tuomela , R. V. Garcia , D. A. Boardman , et al., “TYK2 Inhibition Enhances Treg Differentiation and Function While Preventing Th1 and Th17 Differentiation,” bioRxiv (2024), 10.1101/2024.10.01.616157. PMC12490244 40834860

[46] K. Mine , S. Nagafuchi , S. Akazawa , et al., “TYK2 Signaling Promotes the Development of Autoreactive CD8+ Cytotoxic T Lymphocytes and Type 1 Diabetes,” Nature Communications 15 (2024): 1337, 10.1038/s41467-024-45573-9. PMC10864272 38351043

[47] D. Brandt and C. M. Hedrich , “TCRαβ+CD3+CD4‐CD8‐ (Double Negative) T Cells in Autoimmunity,” Autoimmunity Reviews 17 (2018): 422–430, 10.1016/j.autrev.2018.02.001. 29428806

[48] X. Zheng , J.‐X. Gao , X. Chang , et al., “B7‐CD28 Interaction Promotes Proliferation and Survival but Suppresses Differentiation of CD4‐CD8‐ T Cells in the Thymus,” Journal of Immunology 1950, no. 173 (2004): 2253–2261, 10.4049/jimmunol.173.4.2253. 15294937

[49] R. H. Schwartz , “Natural Regulatory T Cells and Self‐Tolerance,” Nature Immunology 6 (2005): 327–330, 10.1038/ni1184. 15785757

[50] L. Chen and D. B. Flies , “Molecular Mechanisms of T Cell Co‐Stimulation and Co‐Inhibition,” Nature Reviews. Immunology 13 (2013): 227–242, 10.1038/nri3405. PMC3786574 23470321

[51] N. Fortelny , M. Farlik , V. Fife , et al., “JAK‐STAT Signaling Maintains Homeostasis in T Cells and Macrophages,” Nature Immunology 25 (2024): 847–859, 10.1038/s41590-024-01804-1. 38658806 PMC11065702

[52] A. Y. Kreins , M. J. Ciancanelli , S. Okada , et al., “Human TYK2 Deficiency: Mycobacterial and Viral Infections Without Hyper‐IgE Syndrome,” Journal of Experimental Medicine 212 (2015): 1641–1662, 10.1084/jem.20140280. 26304966 PMC4577846

[53] G. Kerner , N. Ramirez‐Alejo , Y. Seeleuthner , et al., “Homozygosity for TYK2 P1104A Underlies Tuberculosis in About 1% of Patients in a Cohort of European Ancestry,” Proceedings of the National Academy of Sciences of the United States of America 116 (2019): 10430–10434, 10.1073/pnas.1903561116. 31068474 PMC6534977

[54] K. Papp , K. Gordon , D. Thaçi , et al., “Phase 2 Trial of Selective Tyrosine Kinase 2 Inhibition in Psoriasis,” New England Journal of Medicine 379 (2018): 1313–1321, 10.1056/NEJMoa1806382. 30205746

[55] R. Al‐Horani , T. Chui , and B. Hamad , “The Pipeline and Market for Psoriasis Drugs,” Nature Reviews. Drug Discovery 23 (2024): 492–493, 10.1038/d41573-024-00018-2. 38297092

[56] J. R. Burke , L. Cheng , K. M. Gillooly , et al., “Autoimmune Pathways in Mice and Humans Are Blocked by Pharmacological Stabilization of the TYK2 Pseudokinase Domain,” Science Translational Medicine 11 (2019): eaaw1736, 10.1126/scitranslmed.aaw1736. 31341059

[57] COVID‐19 Host Genetics Initiative , “Mapping the Human Genetic Architecture of COVID‐19,” Nature 600 (2021): 472–477, 10.1038/s41586-021-03767-x. 34237774 PMC8674144

[58] S. Mocci , R. Littera , S. Tranquilli , et al., “A Protective HLA Extended Haplotype Outweighs the Major COVID‐19 Risk Factor Inherited From Neanderthals in the Sardinian Population,” Frontiers in Immunology 13 (2022): 891147, 10.3389/fimmu.2022.891147. 35514995 PMC9063452

[59] J. Huffman , G. Butler‐Laporte , A. Khan , et al., “Alternative Splicing of OAS1 Alters the Risk for Severe COVID‐19,” MedRxiv (2021), 10.1101/2021.03.20.21254005.

[60] A. Liston , S. Humblet‐Baron , D. Duffy , and A. Goris , “Human Immune Diversity: From Evolution to Modernity,” Nature Immunology 22 (2021): 1479–1489, 10.1038/s41590-021-01058-1. 34795445

