# Supplementary material for: TYK2 :p.Pro1104Ala Variant Protects Against Autoimmunity by Modulating Immune Cell Levels
Source: Immunology. 2025 Jan 21;174(4):462–9. doi: 10.1111/imm.13902 (PMC11885862; doi:10.1111/imm.13902)

**Supplementary material**

**TYK2:p.Pro1104Ala variant protects against autoimmunity by modulating immune cell levels**

Maristella Steri, Valeria Orrù, Carlo Sidore, Antonella Mulas, Maristella Pitzalis, Fabio Busonero, Andrea Maschio, Valentina Serra, Mariano Dei, Sandra Lai, Francesca Virdis, Monia Lobina, Annalisa Loizedda, Michele Marongiu, Marco Masala, Matteo Floris, Nicolò Curreli, Lenuta Balaci, Francesco Loi, Maria Grazia Pilia, Alessandro Delitala, Edoardo Fiorillo, David Schlessinger, Francesco Cucca, Magdalena Zoledziewska

**Supplementary Figure 1.** Regional plot of association of *TYK2*:p.Pro1104Ala variant with published GWAS data on A) T1D, B) RA and C) MS.

A)


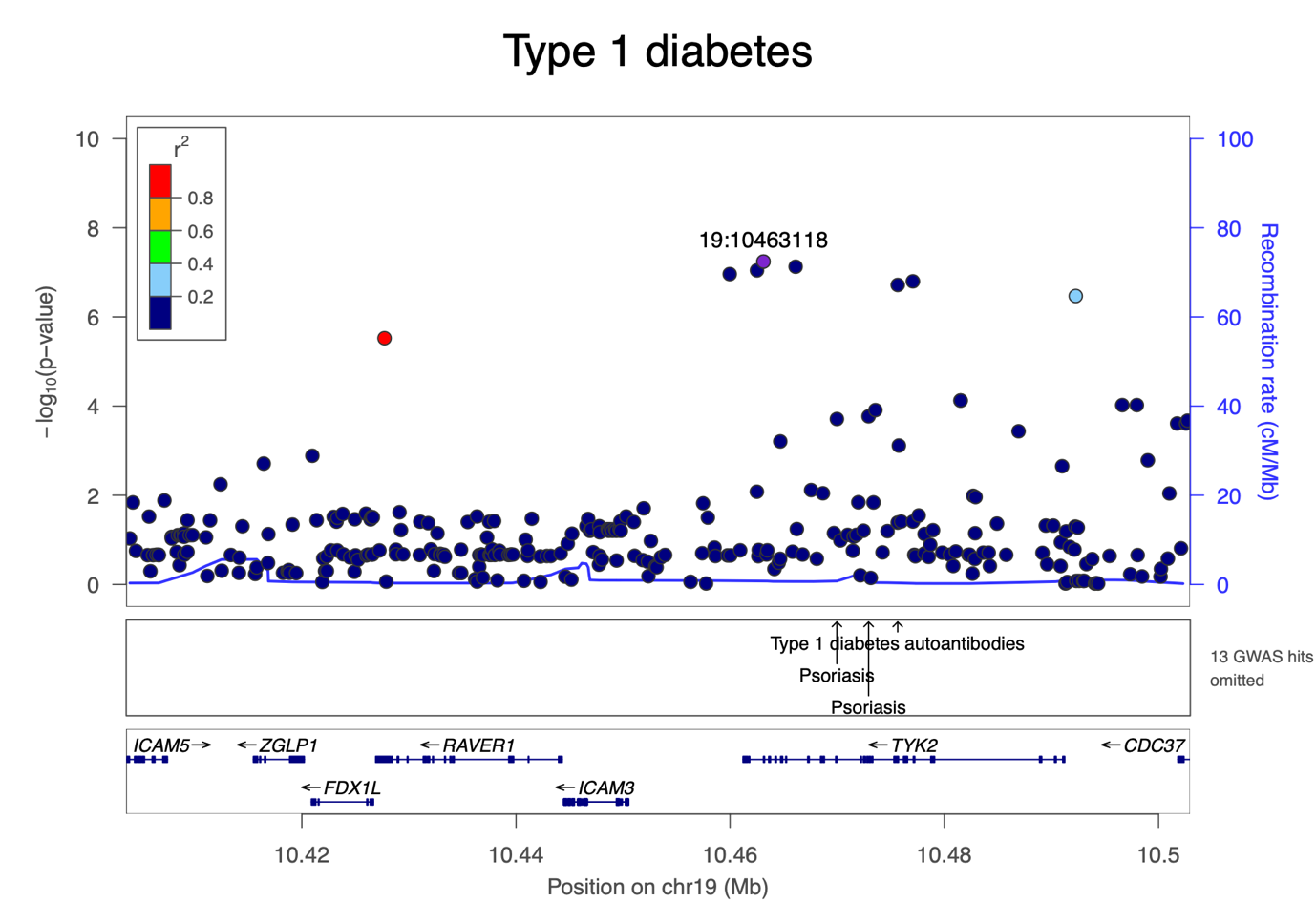


B)


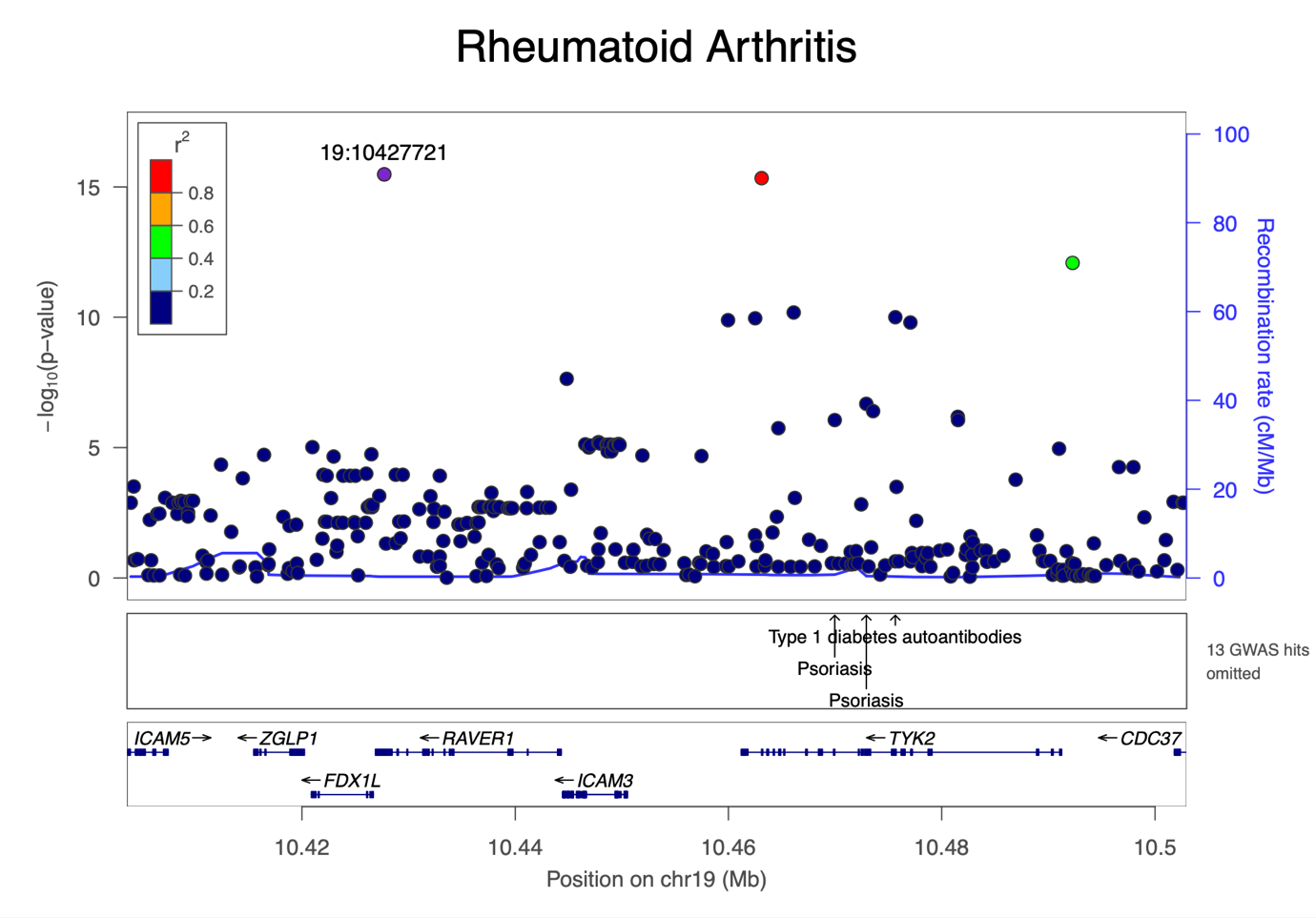


C)


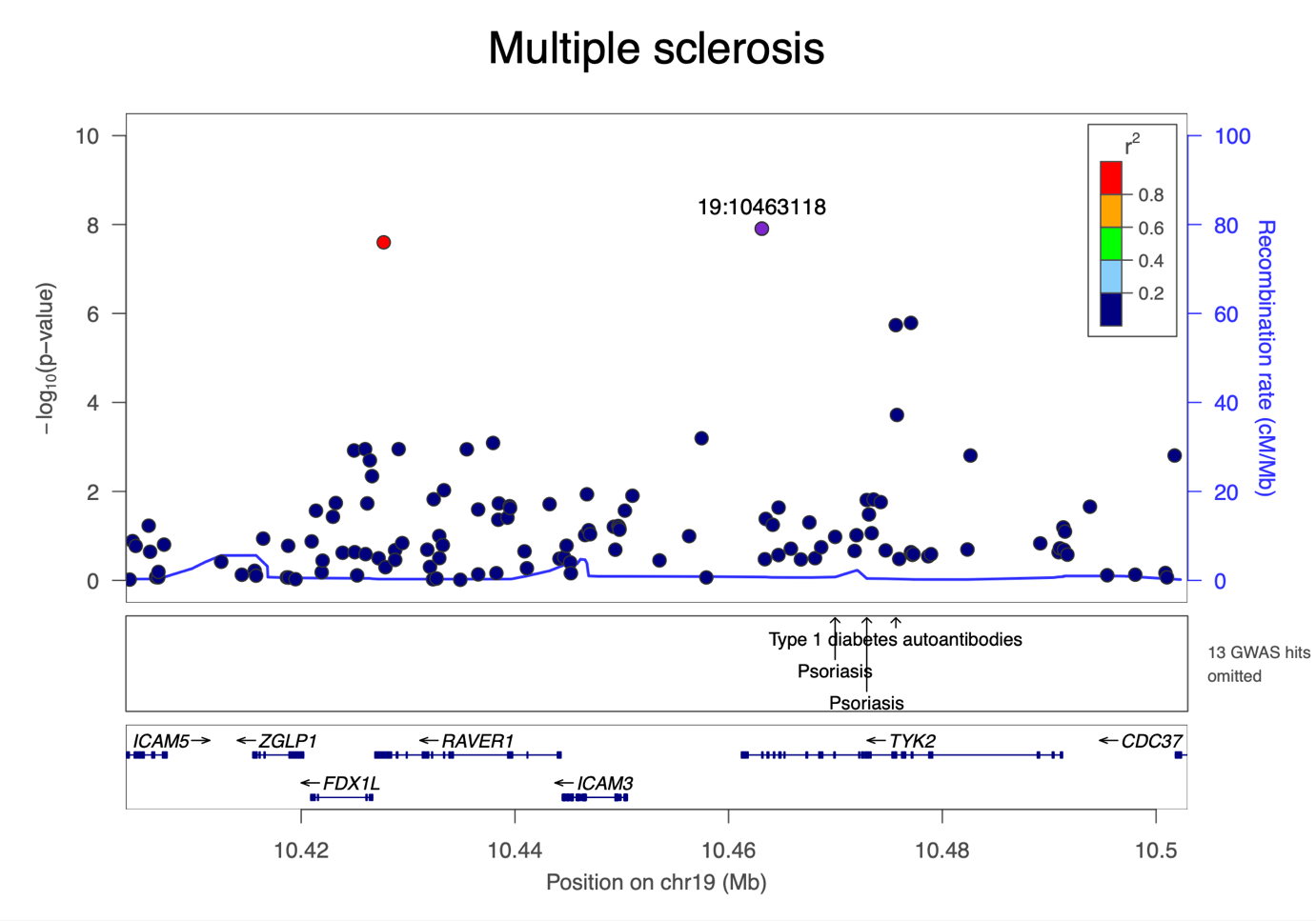


**Supplementary Figure 2.** PheWAS analysis of the associations of *TYK2*:p.Pro1104Ala variant with immune cells levels (p<0.001).


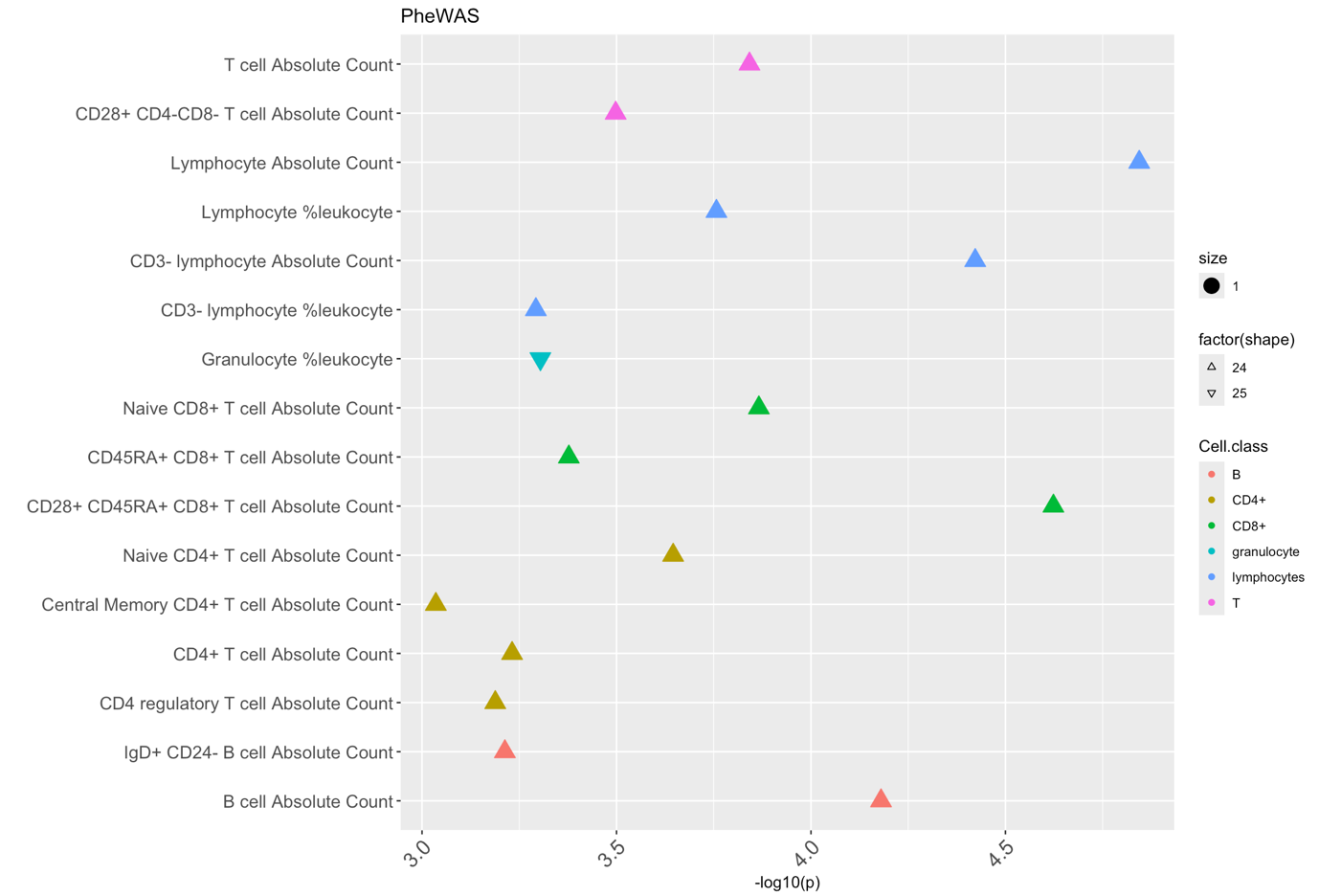

Supplement: Supplementary file 1 — Data S1. [file IMM-174-462-s001.docx]
